# Microbial Culture and Scanning Electron Microscopic Evaluation of Endodontic Hand Files: An In Vitro Study

**DOI:** 10.7759/cureus.25673

**Published:** 2022-06-05

**Authors:** Ahmad H Almehmadi, Faisal T Alghamdi

**Affiliations:** 1 Oral Biology, Faculty of Dentistry, King Abdulaziz University, Jeddah, SAU

**Keywords:** sterilization, cleaning, infection control, reuse of files, endodontic files

## Abstract

Background

Endodontic hand files are used during root canal treatment in the cleaning and shaping step of the procedure. Whether endodontic instruments should be single-use or reusable is a topic of debate. This in vitro study aimed to analyze the bioburden on used and new sterilized endodontic hand files.

Methodology

A total of 30 K-files (15 used, 15 new) and 30 H-files (15 used, 15 new) were studied. After clinical use, the used and new files were subjected to phloxine B staining, scanning electron microscope (SEM) analysis, and microbial culture. We used a Fisher’s exact test to investigate the significant difference in contamination and bioburden between new and used endodontic files.

Results

The chi-square test showed no statistically significant difference between new and used groups in staining. In the used group, 20% of the H-files and 6.7% of the K-files were positive for bioburden (p > 0.05). The SEM analysis showed that all used files (100%) were contaminated with biological debris. All new files and most of the used files (86.7%) were negative for bacterial culture.

Conclusions

Used K-files and H-files (sizes 25 and below) had greater contamination and bioburden than new files. Our results highlight the inadequacy of cleaning methods (mechanical/ultrasonication pre-cleaning and heat sterilization) employed between re-usage of files in this study. Appropriate decision-making on either adapting an evidence-based and effective reprocessing strategy or single-use files can be considered by dentists.

## Introduction

Endodontic files are used during root canals for cleaning and shaping a tooth [1]. Dental instruments are deemed critical when they encounter sterile areas in the body, penetrate the mucosa of the oral cavity, or enter the bloodstream [2]. Endodontic files belong to this category and should be sterilized before use or reuse. Endodontic files are generally considered reusable instruments, but their reusability has been debated. In high-income countries like the United Kingdom, files must be single-use; however, low-income countries allow reuse given their financial constraints [3].

The scrutiny over the reuse of endodontic files centers on the uncertainty of removing biological debris (organic or inorganic) present on the surface of the files following their use in root canals. Biological debris on the files might prevent the penetration of steam. Debris with low moisture content can increase the heat resistance of bacteria and spores [4]. However, the relevance of these findings in modern infection control procedures remains questionable. An in vitro study conducted by Van-Eldik et al. based on microbial evaluation showed that steam sterilization eliminated bacteria in endodontic files irrespective of the type of cleaning procedure used or the presence of biological debris [5]. Phloxine B staining is a simpler, faster alternative to Gram staining and differentiates between Gram-positive and negative bacteria by using visual determination, and it has been utilized in endodontic research [6,7].

Human prion diseases are degenerative disorders involving the nervous system (e.g., Creutzfeldt-Jakob disease; CJD) caused by prion proteins [8]. Biologic debris on instruments may contain prion proteins that are highly infectious and highly resistant to inactivation due to their ability to survive in higher temperatures. Dental pulp can carry prion proteins due to its development from neural crest cells, and the pulp of CJD-infected individuals may be highly infectious [9]. Currently, there is no evidence to suggest the risk of CJD transmission in dentistry [10,11]. However, a lack of clear evidence does not rule out the possibility of disease transmission through biological debris on reused files, and it warrants adequate processing measures through validated methods or manufacturer instructions prior to reuse [12,13]. The Australian and New Zealand Academy of Endodontists stated that they do not have sufficient evidence to restrict endodontic files to single-use [14]. A study conducted in 27 South African dental practices showed that financial constraints were the primary reason that deterred the single-use of endodontic files, and several practices reused the files using different decontamination methods [15]. A knowledge, attitude, and practice survey conducted among 102 dentists and specialists in Saudi Arabia regarding the sterilization of used endodontic files concluded that the general dental practitioners were not following the basic sterilization protocols compared to their counterparts with master’s degrees [16]. The reuse of endodontic files warrants further attention depending on the geographic location and the guidelines for best practices in infection control.

Endodontic files and reamers have a unique design where their fluted and twisted sections can retain bioburden following mechanical or chemical cleaning methods. A study conducted by Ferreira Murgel et al. showed that none of the cleaning techniques such as ultrasonic bath, gauze with alcohol, and sponge with alcohol effectively removed the debris from both used and unused files. The cleaning techniques did not eliminate the debris used immediately or one hour after the canal preparation [17]. Another study conducted by Bryson et al. concluded that cleaning methods using a washer-disinfector, ultrasonic bath, and ultrasonic bath with cavitation did not effectively remove the residual protein from rotary nickel-titanium (Ni-Ti) files [18]. Smith et al. performed a light microscopic evaluation of endodontic files received from the hospital and general dental setting following cleaning using a hand brush or ultrasonic bath. The files from dental practice were cleaned using a hand brush, and 76% retained biological debris. Moreover, the files from the hospital setting were cleaned using an ultrasonic bath, but only 14% had bioburden [19]. Even though many studies have assessed the sterility of the endodontic files subject to reuse, there is still a lack of evidence on culture and scanning electron microscopy (SEM) analysis in a high-income country like Saudi Arabia [20]. This in vitro study aimed to evaluate the bioburden on sterilized new and used endodontic hand files using methods such as bacterial culture, phloxine-B staining, and SEM analysis.

## Materials and methods

We conducted this in vitro study at the Faculty of Dentistry at King Abdulaziz University. The study protocol was approved by the Research Ethics Committee of the Faculty of Dentistry at King Abdulaziz University (No. 92-08-20). No informed consent was required in this type of study. The present study complied with the principles of the Declaration of Helsinki. This in vitro study included 60 endodontic hand files (30 K-files, 30 H-files), with an equal number of new and used files (Table 1). We used size 25 files; the K-files were 21 mm, and the H-files were 25 mm. The files were collected from different private and governmental dental schools in Saudi universities in Jeddah, Saudi Arabia, where postgraduate students performed root canal treatment. We used a simple random sampling technique.

**Table 1:** Distribution of H-files and K-files among the used and new groups.

Study samples	H-Files N (%)	K-Files N (%)	Total N (%)
Used	15 (25)	15 (25)	30 (50)
New	15 (25)	15 (25)	30 (50)
Total N (%)	30 (50)	30 (50)	60 (100)

The cleaning and shaping of the root canals were performed using these files with 15 mL of 3% sodium hypochlorite solution (NaOCl) delivered with the EndoVac® irrigation system (Vista Dental, Racine, WI, USA) for five minutes in each canal and using a 30-gauge, side vented irrigating needle (Max-I-Probe, Dentsply Maillefer, Ballaigues, Switzerland). The files were placed in a sterile container with distilled water for two weeks until the used files were collected, and the water was replaced every week. The files were observed under a stereoscopic microscope with 40× magnification to rule out any instruments with fracture or deformation.

Sterility was maintained during procurement via nitrile gloves and mosquito forceps for catching instruments. Moreover, 30 new files (15 K-files, 15 H-files) were procured of the same size and length as the used files to serve as controls. All the endodontic files were subjected to manual and ultrasonic cleaning performed following the same protocol used in Cayo-Rojas et al.’s study [21]. All the investigated endodontic files were mechanically pre-cleaned for 30 seconds with a nylon brush (Oral B Pro Salud, Procter & Gamble, Cincinnati, OH, USA) and enzymatic detergent (Alkazyme, Alkapharm, Romainville, France) and then soaked in the enzymatic detergent for 15 minutes. Samples were then brushed once again, gripping the handle firmly and spinning each H- and K-type file to brush all of its flutes and cutting surfaces, replacing the brush after every 15 H- and K-type file. Samples were then washed for 15 seconds with distilled water and dried with sterile gauze. Regarding the ultrasonication, we employed the same enzymatic solution and rinsed for 15 minutes under running distilled water which served as the basis for effective cleaning protocols before the ultrasonic cleaner [22]. Samples were then washed with distilled water and inserted into the ultrasonic equipment (Pro Ultrasonic Cleaner, Digitec Galaxus AG, Zürich, Germany), as recommended by the manufacturer. After that, the H- and K-type files were cleaned with distilled water and dried with sterile gauze. The sample size calculation was performed per Van Eldik et al.’s study with 95% power and 5% alpha error [5].

Phloxine-B staining and SEM

**Figure 1:**
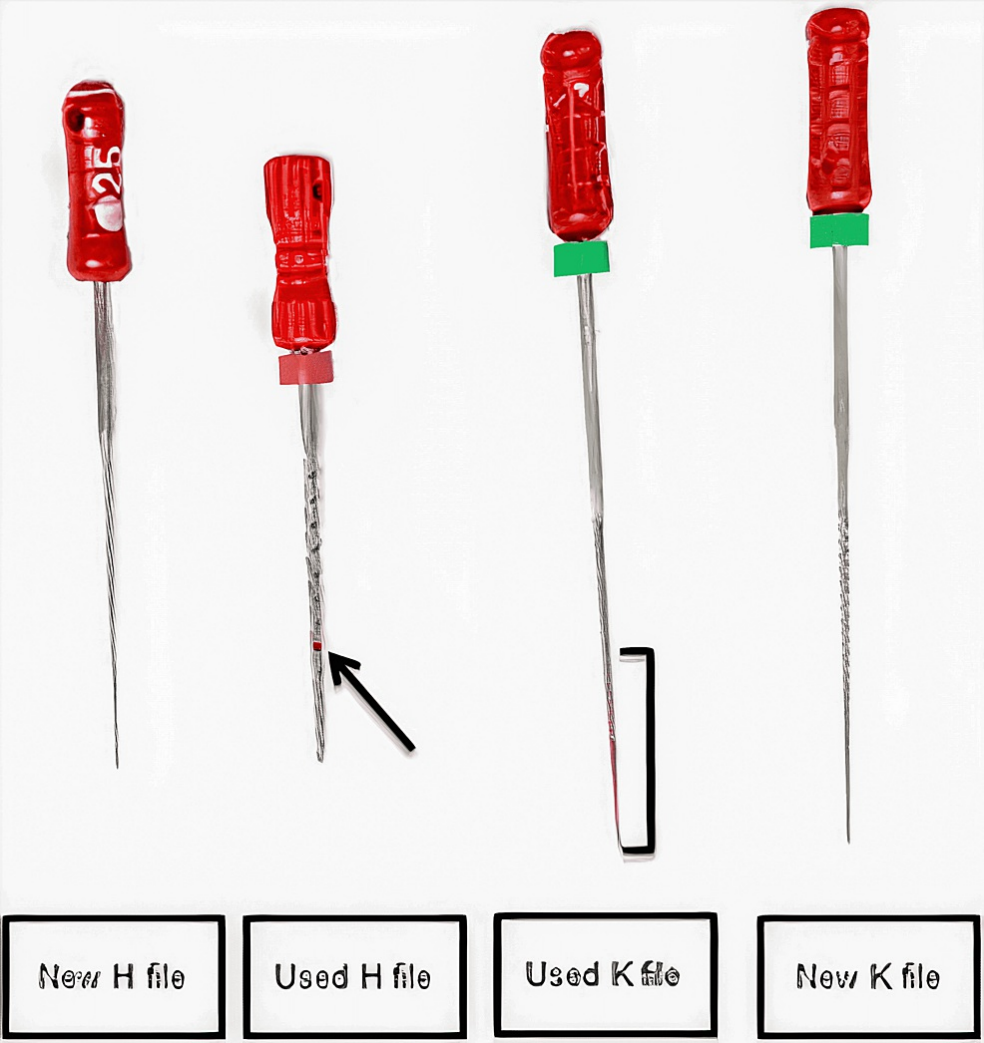
Staining endodontic files with phloxine-B. Both new and used sterilized files were stained with phloxine-B and rinsed with distilled water. Note the red staining on the used K-file (right bracket) and the used H-file (arrow) compared to no staining on new K-files and H-files.

**Figure 2:**
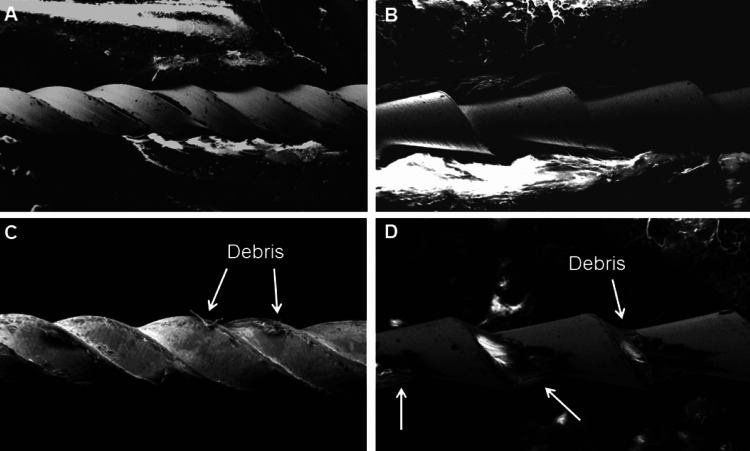
SEM of new versus used endodontic files. Note clear surfaces of files with no debris in (A) new K-file and (B) new H-file. Note clear surfaces of files with debris (pointed by arrows) in (C) used K-file and (D) used H-file. SEM: scanning electron microscope

We used a two-step experimental protocol in this in vitro study. The used and new instruments were stained with phloxine-B and photographed. Staining was performed by placing each file in a plastic container with 2 mL of phloxine-B, and the sealed plastic bag was placed in an ultrasonic bath for 15 minutes. The files were removed from the bags, washed in deionized water, and air-dried. We observed the files using an oblique light, and the camera (Cannon t31 Rebel) zoomed in on the flutes and cutting surfaces (Figure 1) [23,24]. The photographs were uploaded to a computer with 40× magnification and an accelerating voltage set at 15 kV. The bioburden on the files was observed using SEM (Zeiss Q150R, Germany) at the flutes and cutting surfaces (Figure 2). Two examiners analyzed the photographs and SEM images.

We assessed the inter-examiner reliability of the SEM images via a pilot study. The inter-examiner reliability was performed using kappa statistics, and the kappa value was 0.76, indicating a substantial level of agreement between the examiners. Construct validity was investigated using the Spearman rank correlation coefficient. The SEM photographs were moderately correlating with the bacterial culture (gold standard) with a Spearman correlation rho value of 0.667.

Microbial analysis

**Figure 3:**
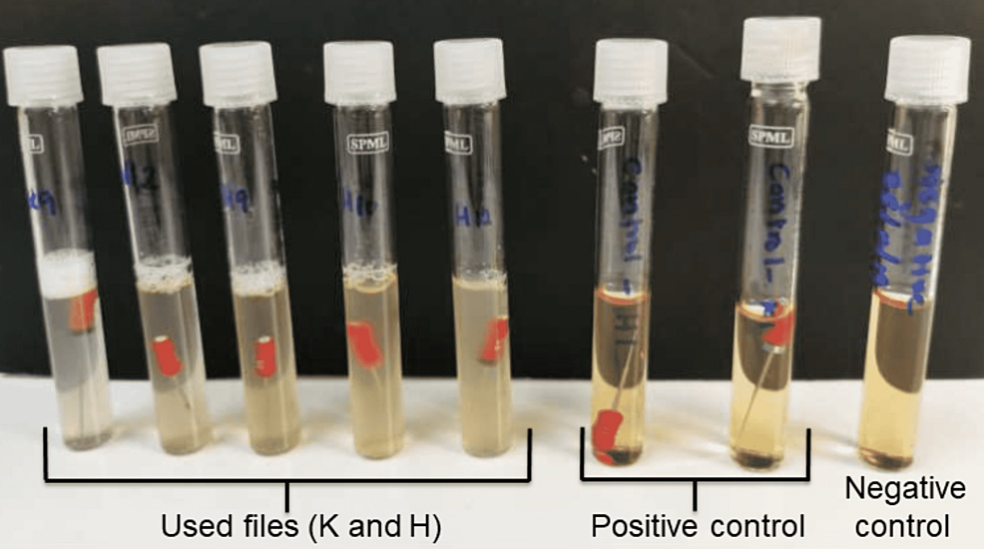
Culture of new and used sterilized endodontic files in 10-mL tubes of BHI broth. Turbidity was observed in all used sterilized endodontic K-files and H-files compared to clear control tubes containing only broth as a negative control and new K-files and H-files as positive controls. BHI: brain-heart infusion

All the new and used files were subjected to bacterial culture, with the instruments being aseptically placed in sterile test tubes containing 10 mL brain-heart infusion (BHI) broth. BHI is a growth medium specifically used for fastidious organisms. A negative control with an additional test tube containing only 10 mL BHI and a positive control using sterilized new H- and K-files were established for this culture. All the tubes were subjected to culture for 10 days at 37°C in a 5% carbon dioxide milieu. The presence of bacterial colonies was assessed by checking the turbidity in the test tubes (Figure 3).

**Figure 4:**
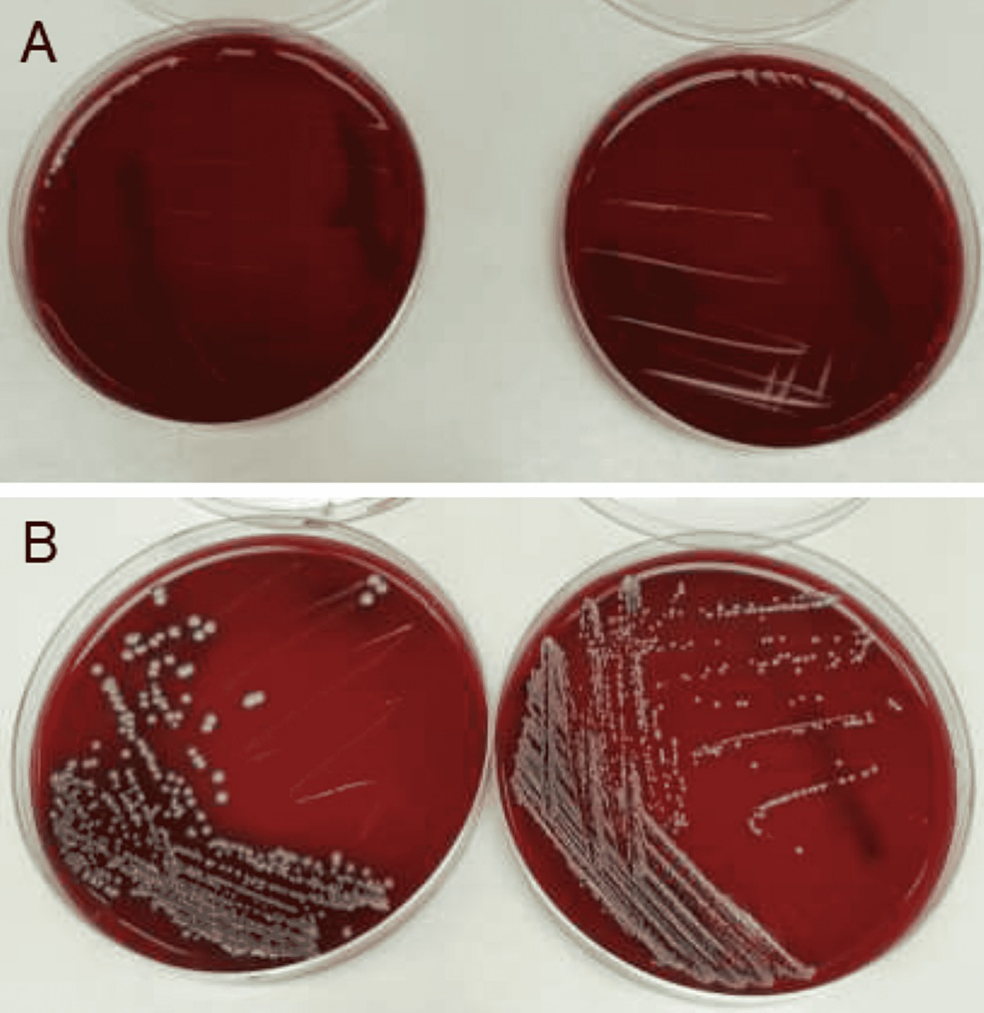
Culture dishes of used and new files. Representative culture dish showing (A) no growth in new K-files and H-files and (B) bacterial growth in used K-files and H-files.

We tested an inoculum obtained from the test tubes for turbidity, and it was added to Petri dishes containing BHI and potato dextrose agar (PDA), a general-purpose medium for yeasts and molds. The BHI Petri dishes were cultured at 37°C in 5% carbon dioxide for up to 48 hours. The Petri dishes with the inoculum were cultured at 30°C for five days, and we repeated the process to obtain a pure culture of bacterial colonies. Subsequently, they were stored at -80°C in 20% glycerol (Figure 4). The characteristics and identity of the bacterial colonies were analyzed using Gram staining and biochemical tests (i.e., catalase test and growth in an alkaline medium; pH of 9.2). We used an automated identification system (VITEK 2, bioMérieux SA, Marcy-l'Étoile, France) to identify the bacteria rapidly. The fungal growth observed in the Petri dishes was subjected to multiple cultures using three media, PDA, Malt extract agar, and Capek’s solution, for seven days at 30°C until pure cultures were obtained and stored. The fungal identification was based on morphology, appearance, and coloration.

Statistical analysis

The data were analyzed using SPSS Statistics for Windows, version 25.0. (IBM Corp., Armonk, NY, USA). Data were subjected to statistical analysis in form of descriptive statistics, percentages, and frequencies. We used a Fisher’s exact test to investigate the significant difference in contamination and bioburden among used and new files, and we considered p ≤ 0.05 as statistically significant.

## Results

Among the used files, 80% of the H-files and 93.3% of the K-files were negative for bioburden, while all new files had no evidence of bioburden in the staining experiment (p > 0.05; Table 2). Among used files, 20% of the H-files and 6.7% of the K-files were positive for bioburden in the staining experiment, but the difference was not statistically significant (p > 0.05; Table 3). The SEM images indicated that all used files were contaminated with biological debris regardless of type. However, all new files (both types) were free of biological debris, and the difference between groups was statistically significant (p < 0.05; Table 4).

**Table 2:** Distribution of phloxine-B staining in all H-files and K-files. P < 0.05 considered statistically significant.

Phloxine-B staining	H-Files, n (%)		K-Files, n (%)	
	Negative	Positive	Negative	Positive
Used	12 (80.0)	3 (20.0)	14 (93.3)	1 (6.7)
New	15 (100)	0 (0)	15 (100)	0 (0)
P-value	0.068		0.309	

**Table 3:** Distribution of phloxine-B staining among the used H-files and K-files. P < 0.05 considered statistically significant.

Phloxine-B staining	Used files, n (%)	
	Negative	Positive
H-Files	12 (80.0)	3 (20.0)
K-Files	14 (93.3)	1 (6.7)
P-value	0.283	

**Table 4:** Distribution of SEM imaging in H-files and K-files. *P < 0.05 considered statistically significant. SEM: scanning electron microscope

SEM imaging	H-Files, n (%)		K-Files, n (%)	
	Negative	Positive	Negative	Positive
Used	0 (0)	15 (100)	0 (0)	15 (100)
New	15 (100)	0 (0)	15 (100)	0 (0)
P-value	0.001*		0.001*	

Most of the used H-files (66.7%) and K-files (86.7%) and all the new H-files and K-files (100%) tested negative for bacterial contamination, and the difference was statistically significant (p < 0.05; Table 5). Significantly fewer K-files (13.3%) tested positive for bacterial contamination than H-files (33.3%; chi-square, 62.354; p < 0.05; Table 6). The bacterial species identified from used K-files include *Staphylococcus aureus *(*S. aureus*) and *Klebsiella pneumonia* (*K. pneumonia*). Used H-files yielded *Staphylococcus lugdunensis* (*S. lugdunensis*) and *Staphylococcus hominis* (*S. hominis*).

**Table 5:** Distribution of bacterial contamination in all H-files and K-files. *P < 0.05 considered statistically significant.

Bacterial contamination	H-Files, n (%)		K-Files, n (%)	
	Negative	Positive	Negative	Positive
Used	10 (66.7)	5 (33.3)	13 (86.7)	2 (13.3)
New	15 (100)	0 (0)	15 (100)	0 (0)
P-value	0.001*		0.001*	

**Table 6:** Distribution of bacterial contamination among used H-files and K-files. *P < 0.05 considered statistically significant.

Bacterial contamination	Used files, n (%)	
	Negative	Positive
H-Files	10 (66.7)	5 (33.3)
K-Files	13 (86.7)	2 (13.3)
P-value	0.001*	

## Discussion

We tested 60 new and used endodontic files for microbial contamination and bioburden. There is evidence in the recent literature recommending single use of endodontic files due to difficulty in cleaning, no universal cleaning standard, risk of transmission of infectious diseases, and corrosion/dulling of endodontic files [15]. The single-use nature of endodontic files is debatable, given that the disposal of rotary endodontic instruments poses a considerable economic burden on the dentist and the patient [14]. Also, a study conducted by Parashos et al. involved cleaning used endodontic files using three stages of mechanical and chemical cleaning. It concluded that the cleaning protocol showed 100% effectiveness and Ni-Ti files were devoid of stained debris, and it questioned the recommendation of single-use endodontic files [25]. A study of 150 files obtained from six manufacturers found that 13% of files tested positive for bacterial colonies and concluded that even new files should undergo sterilization before clinical usage [26].

Hand instruments, if reused, are reused approximately five to ten times and are discarded due to structural damage [27]. This study assessed the bioburden on used endodontic files compared to new files using phloxine B staining. Phloxine B (acid red 92) is a fluorescein derivative stain used to detect proteins and peptides. Its applications include forensics as a blood staining test and microbial detection [23,28]. It primarily targets the proteinaceous part of the contamination on the endodontic files. Proteins can get degraded to tenacious biological moieties known as prions that can retain their infective potential over a considerable period [29].

To prevent any contamination to occur during the experiment, the sample collection was performed in a strict sterilized manner, as mentioned in the Materials and Methods section. In addition, all the positive and negative control samples confirmed the samples were free from any contamination may occur during the microbial and SEM analysis and confirmed the validity of the findings. We saw that 20% of used H-files and 6.7% of used K-files were positive for biological debris, whereas the new files had no contamination. Differences between file types and the used or new status were not statistically significant, which might be attributable to the small sample size in this study or the possibility of NaOCl used in cleaning and shaping that may have denatured the proteins. Bryson et al. observed biological contamination in 200 used rotary Ni-Ti files following pre-sterilization cleaning using an ultrasonic bath and ultrasonic bath acoustic cavitation by staining with Van Gieson’s solution. Bryson et al. concluded that none of the two methods could altogether remove the bioburden on the clinically used files following the staining experiment. These reagents can only stain proteins, they cannot detect prions, and the biological debris may not necessarily pose a risk for CJD [18]. The staining results may be a preliminary finding, and future research can explore the protein components with robust and elaborate detection assays to verify the results [30].

The SEM results of this in vitro study showed that all the used files were positive for biological debris and the new files were free of debris. Buchanan et al. studied 401 endodontic hand files from 27 dental practices in South Africa. They used a stereomicroscope to search for biological debris remnants following reprocessing of instruments. Two calibrated examiners concluded that 94% of the used files were contaminated with debris [15]. Our findings also agree with two studies that observed that 98% and 96% of study samples were contaminated with biological debris [31,32]. Other studies have reported visual evidence of debris contamination in endodontic files following decontamination protocols in routine practice [19,31,33].

Our microbial analysis revealed that new files were free of bacterial or fungal contamination, as were 66.7% of used H-files and 86.7% of used K-files. The differences in the bacterial contamination levels may be attributable to the number of threads, flute design, and shape of cutting surfaces between the two types of files. Van Eldik et al. reported that size 35 H-files retained more bacteria than smaller files. They found a slight variation in the bacterial retention based on the type of file compared with rotary files [5]. We saw more bacterial contamination in the H-file than in the K-file, possibly due to the greater working surface area on the H-file that may provide a niche for debris. Based on our results and those reported by Van Eldik et al. [5], the file type can influence the bioburden present on the files.

In this study, the most common microbial species isolated from the files were *S. aureus*,* S. lugdunensis*,* S. hominis*, and* K. pneumoniae*. *Staphylococcus spp*. (Gram-positive) have been associated with angular cheilitis, parotitis, periodontitis, and a unique condition known as staphylococcal mucositis [34]. *S. aureus*,* Enterococcus faecalis*, and *Candida albicans* are the most resistant microorganisms in infected root canals and are associated with failed root canal treatment [35,36]. Pathogenic microorganisms were isolated from used files following sterilization, which warrants further analysis with assessment of microbial load and larger sample sizes to ascertain its clinical relevance.

Regarding the removal of biologic waste according to endodontic file size when using both cleaning methods, Nosouhian et al. [37] discovered a greater amount of biologic waste in the smallest K-type size files (#15 and #25), agreeing with the present findings as the H- and K-type files with the highest level of biologic waste after the cleaning methods were the used H- and K-type files, size #25. However, these findings must be considered with caution because the interquartile range for both cleaning methods is high in all the bacterial contamination samples of used H- and K-type file groups by (five samples -33.3%) and (two samples - 13.3%), respectively.

Both the mechanical and ultrasonic cleaning methods were significantly more effective when enzymatic detergent was used to clean H- and K-type files, though it is clear that neither method completely removed biologic waste from all used H- and K-type files, which might contribute to inadequate sterilization of endodontic files and, as a result, be a point to be considered in clinical cross-infection.

We found five (33.3%) samples with *S. lugdunensis *and *S. hominis* in the used H-file group. There are no published reports on *S. lugdunensis* and *S. hominis* as part of the root canal microbiota. Given that we found *S. lugdunensis*, *S. hominis*, and several species of *the Staphylococcus* family as prevalent microbes residing on endodontic files, future studies are necessary to determine the many types of microorganisms that can be detected among different endodontic instruments. Our study was the first of its kind conducted in Saudi Arabia. However, the study was limited by its small sample size, and the results should be explored further by including the effect of cleaning and decontamination methods commonly employed in routine practice and protein detection assays.

## Conclusions

This in vitro study is a preliminary step to assess the bioburden and microbial contamination on used endodontic files using a combination of phloxine B staining, SEM, and bacterial culture. Despite cleaning measures, used K-type and H-type endodontic files (sizes 25 and below) contained biological debris and bacterial contamination, while new files were free of debris and contamination. Although there are no universal guidelines on the reprocessing of endodontic instruments imposed on dental practices in Saudi Arabia, the oral care community should note our study’s implications that highlight the inadequacy of decontamination procedures (mechanical/ultrasonication pre-cleaning and heat sterilization) utilized in this study. Routine dental practices must maintain the highest infection control standards, and appropriate decision-making in terms of single-use or appropriate evidence-based reprocessing strategy should be utilized.
